# Correlation Between Intracranial Calcification and Extracranial Stenosis of the Internal Carotid Artery

**DOI:** 10.7759/cureus.40234

**Published:** 2023-06-10

**Authors:** Julia R Jahansooz, Andrew Ko, Ryoko Hiroi, Masako Matsunaga, Enrique Carrazana, Jason Viereck

**Affiliations:** 1 Neurology, John A. Burns School of Medicine, Honolulu, USA; 2 Brain Research, Innovation & Translation Laboratory, Hawaii Pacific Neuroscience, Honolulu, USA; 3 Quantitative Health Sciences, John A. Burns School of Medicine, Honolulu, USA

**Keywords:** carotid artery disease, vascular calcification, ischemic stroke, internal carotid artery, extracranial stenosis, intracranial calcification

## Abstract

Intracranial artery calcification is a marker of vascular atherosclerosis and has a high prevalence worldwide. Both atherosclerosis of the internal carotid artery at the carotid sinus in the neck and intracranial calcification have been associated with ischemic stroke. The relationship between the two has not been well studied. The present study investigated how carotid sinus narrowing could relate to calcification located in the distal intracranial artery at the cavernous carotid. We examined a population not selected for cerebral disease. This retrospective study contained 179 subjects aged 18 years and older from the Hawaii Diagnostic Radiology database. Extracranial internal carotid artery stenosis was determined using the absolute diameter, North American Symptomatic Carotid Endarterectomy Trial, and common carotid artery methods. Calcification was scored using the modified Woodcock method. A positive correlation between intracranial calcification and extracranial carotid stenosis was found using all three methods. Individuals with intracranial calcification were more likely to be older, have a smaller internal carotid artery diameter, and have a greater percent stenosis at the internal carotid artery than those without intracranial artery calcification (p<0.001 for all). These results may help refocus interest in calcification in studies of cerebral vasculature and its correlation with extracranial carotid stenosis.

## Introduction

This article was previously presented as an oral presentation at the 2022 Annual Biomedical Sciences & Health Disparities Symposium on April 7, 2022.

In 1954, C. Miller Fisher published the results of 432 autopsies showing a 9.5 percent prevalence of carotid artery disease with 45 patients having either uni- or bi-lateral occlusion of their carotid arteries [1]. Several more cases had non-complete occlusion of the carotid arteries contributing to the identification of internal carotid artery (ICA) atherosclerosis as a major cause of ischemic stroke [2].

Calcification is an active component of atherosclerosis and can be found in up to 90 percent of atherosclerotic lesions [3-5]. In smaller elastic vessels such as the intracranial carotid arteries, calcification typically develops in the intimal and medial layers of blood vessels [6]. The presence of intimal calcification can lead to the formation of atherosclerotic plaques leading to vessel stenosis. Studies conducted by O’Rourke et al. established a link between coronary artery calcification and coronary disease [7]. This precipitated growing research in investigating the predictive values of vascular calcification in other arteries.

Vascular intracranial calcification is typically associated with intracranial atherosclerosis, cavernous angioma, arteriovenous malformation, or an aneurysm [8]. The pathophysiology between extracranial ICA stenosis and intracranial ICA calcification is not well understood. Within the intracranial arteries, the ICA is the most common site of calcification in stroke patients [5]. Incidence of intracranial ICA calcification can range from 60-90 percent depending on age, presence of stroke, and other risk factors. Previous studies have identified a link between intracranial artery calcification and stroke, although not consistently [3,5,9-15].

Correlations have previously been identified between calcification and atherosclerosis within the same arterial segment. For example, a study by Kim et al. found that calcification of the cavernous portion of the ICA reflected the overall cerebral atherosclerosis burden in patients with an acute cerebral infarction [16]. A similar study by Baradaran et al. identified a weak correlation between intracranial luminal stenosis and intracranial calcification which in this context refers to calcifications within the ICA [17]. However, the only study we are aware of linking stenosis to more distal calcification was a study by C. Miller Fisher. He showed a correlation between severe intracranial calcification at the carotid siphon and advanced atherosclerosis of the ICA at the carotid sinus [17,18]. We hypothesized that there is a positive correlation in our population of subjects.

Stenosis at the proximal ICA can be calculated by different methods [19]. The North American Symptomatic Carotid Endarterectomy Trial (NASCET) method is the most widely used in literature and is clinically useful. However, it only provides relative stenosis and does not consider the absolute residual diameter of the lumen. C. Miller Fisher felt absolute diameter was most important in pathophysiology [1]. Computed tomography angiograms (CTA) now provide the ability to precisely measure residual lumen unlike conventional arteriograms [20]. Analysis using common carotid artery (CCA) methods is included as this is also represented in the literature [19]. To thoroughly explore the hypothesized relationship, we used all three methods of stenosis measurement.

## Materials and methods

Inclusion criteria and imaging

This retrospective study analyzed head and neck computed tomography (CT) scans of 179 subjects aged 18 years and older from the state of Hawaii. Imaging was performed at Hawaii Diagnostic Radiology (https://www.hawaiidrs.com/), a community neuroradiology practice that represents a diverse population of patients from various medical centers throughout Oahu. If the modified Woodcock scores and/or the left and right carotid diameters could not be measured in the scan, the subject was retroactively excluded from the study. This research project has been approved by the Institutional Review Board at the University of Hawaii as exempt approved research (protocol number is 2019-00837). Informed consent was not required for inclusion in this minimal risk, retrospective study and its evaluation of records and images.

CT images used in this study include CT soft tissue neck with contrast (STN W), CT soft tissue neck without contrast (STN WO), CT soft tissue neck with and without contrast (STN WOW), CTA neck with contrast (N W), CTA neck with and without contrast (N WOW), and CTA head and neck with and without contrast (HN WOW). A study by Saade et al. noted that intracranial calcifications are commonly encountered in non-contrast CT studies and are the imaging modality of choice for characterization of calcifications [8]. All CT scans were 2.5 millimeters in diameter. CT images were scanned using a Discovery CT750 HD CT scanner. Merge Unity software was used for evaluation of stenosis and the built-in software for assessing calcium was GE AW: Smartscore software version 4.0.

Evaluation of CT scans

To determine the degree of extracranial ICA stenosis, absolute diameter, NASCET, and CCA methods were used. Three measurements were recorded for analysis following the methodology noted by Rothwell et al. [20]. CT scans of the neck were identified and visualized beginning at the inferior portion of the scan and moved superiorly until the ICA bifurcation. Absolute diameter in millimeters was measured prior to the carotid bifurcation, immediately following the carotid bifurcation, and distal to the carotid bifurcation. Data collection was conducted bilaterally using the measurement tool found within the imaging software.

The CT bone window was used for the intracranial calcification measurements following the modified Woodcock visual scoring method [16]. The cavernous carotid artery was identified using the dorsum sellae and posterior clinoid process as landmarks. Starting inferiorly, the carotid artery was followed superiorly until the circle of Willis. Calcification measurements were not recorded at or beyond this point. A slice was assigned a score of 0 for no calcification, a 1 for thin, discontinuous calcification, a 2 for thin, continuous OR thick, discontinuous calcification, or a 3 for thick, continuous calcification. The individual slice scores were combined and recorded as the overall calcium score for that artery.

Statistical analyses

Characteristics of the study subjects (n=179) were described by the mean (standard deviation), median (interquartile range), range for numeric variables, and the frequency (percentage) for categorical variables. Characteristics of the intracranial calcification groups (adult patients with and without intracranial artery calcification) were compared by Wilcoxon rank-sum tests for numeric variables and Pearson’s Chi-squared tests for categorical variables. The monotonic relationships between modified Woodcock scores and degrees of stenosis (absolute ICA diameters, CCA ratios, and NASCET ratios) among adult patients were examined by Spearman’s rank correlation tests. A p-value <0.05 was considered statistically significant for hypothesis testing, and all analyses were conducted in R version 4.0.2.

Prior to data analysis, 52 records were excluded due to the unavailability of their modified Woodcock score. Seven records were not analyzed because both their left and right carotid absolute diameters were unavailable and four records were excluded due to the participant being under the age of 18.

**Figure 1:**
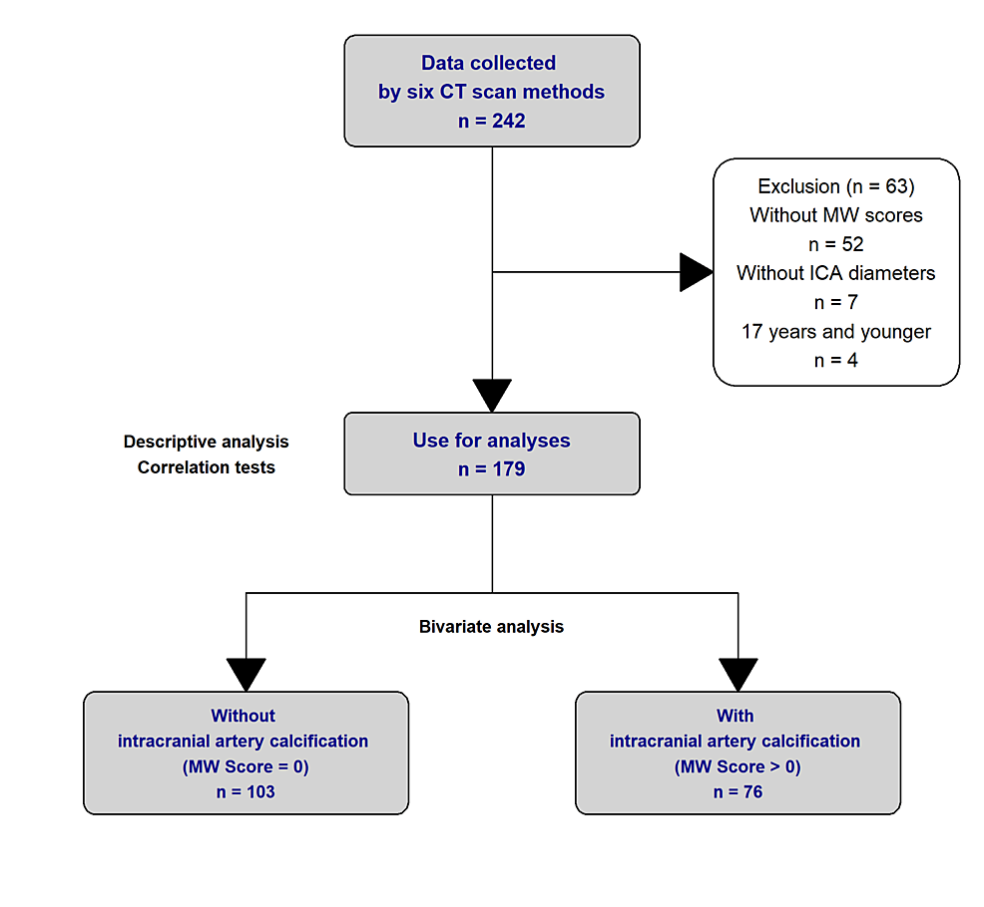
Analysis Breakdown

A total of 179 records from 179 unique individuals were included in the data analysis (Figure 1). During analysis, the more severe measurement of extracranial ICA stenosis between the left and right values was used. The modified Woodcock scores from both the left and right intracranial ICA were combined prior to analysis.

## Results

Relationship between extracranial ICA diameters and intracranial ICA calcification measurements

Table 1 displays the demographic and analytical data derived from this study. The average age of the participants was approximately 59.4 years old with 51.4 percent of participants being male. A significant difference in levels of stenosis and calcification was found among age groups with those over the age of 68 having calcification more often than those in the 55-68 or under 55 age groups (p<0.001). There was no significant difference found among sex (p=0.46). Intracranial calcification was present in 40.18 percent of individuals and the average total calcification score was 3.4. The average absolute diameter, CCA ratio, and NASCET ratio of an individual’s extracranial carotid artery were 6.1 mm, 15.2 percent, and 6.0 percent, respectively.

**Table 1:** Descriptive Data of Extracranial ICA Stenosis and Intracranial ICA Calcification ICA: Internal carotid artery; CCA: Common carotid artery; NASCET: North American Symptomatic Carotid Endarterectomy Trial

	Overall, N = 179	Intracranial Artery Calcification		p-value^1^
		Without, N = 103 (58%)	With, N = 76 (42%)	
Age				<0.001
Mean (SD)	59.4 (17.0)	51.2 (15.6)	70.5 (11.7)	
Median (IQR)	60.0 (51.0, 70.0)	54.0 (37.0, 63.0)	70.0 (62.0, 79.0)	
(33%, 66%)	(54.0, 68.0)	(44.3, 58.3)	(64.8, 77.5)	
Range	19.0, 94.0	19.0, 84.0	39.0, 94.0	
Age Group, n (%)				<0.001
<55	60 (33.5%)	54 (52.4%)	6 (7.9%)	
55-68	62 (34.6%)	35 (34.0%)	27 (35.5%)	
>68	57 (31.8%)	14 (13.6%)	43 (56.6%)	
Sex, n (%)				0.46
Male	91 (51.4%)	50 (49.0%)	41 (54.7%)	
Female	86 (48.6%)	52 (51.0%)	34 (45.3%)	
(missing)	2	1	1	
MW score				<0.001
Mean (SD)	3.4 (7.91)	0.0 (0.00)	8.1 (10.52)	
Median (IQR)	0.0 (0.00, 4.0)	0.0 (0.00, 0.0)	5.0 (2.00, 10.0)	
Range	0.00, 76.0	0.00, 0.0	1.00, 76.0	
ICA diameter at Location A				<0.001
Mean (SD)	6.1 (1.86)	6.5 (1.35)	5.4 (2.22)	
Median (IQR)	6.4 (5.10, 7.3)	6.6 (5.90, 7.3)	5.8 (3.90, 6.9)	
Range	0.00, 9.8	2.60, 9.6	0.00, 9.8	
CCA ratio (%)				<0.001
Mean (SD)	15.2 (22.20)	7.9 (13.03)	25.0 (27.73)	
Median (IQR)	4.5 (0.00, 21.9)	0.0 (0.00, 12.3)	15.5 (0.00, 45.9)	
Range	0.00, 100.0	0.00, 62.3	0.00, 100.0	
NASCET ratio (%)				<0.001
Mean (SD)	6.0 (17.99)	1.2 (6.47)	12.6 (25.19)	
Median (IQR)	0.0 (0.00, 0.0)	0.0 (0.00, 0.0)	0.0 (0.00, 10.7)	
Range	0.00, 100.0	0.00, 52.5	0.00, 100.0	
^1 ^Wilcoxon rank sum test; Pearson's Chi-squared test				

**Figure 2:**
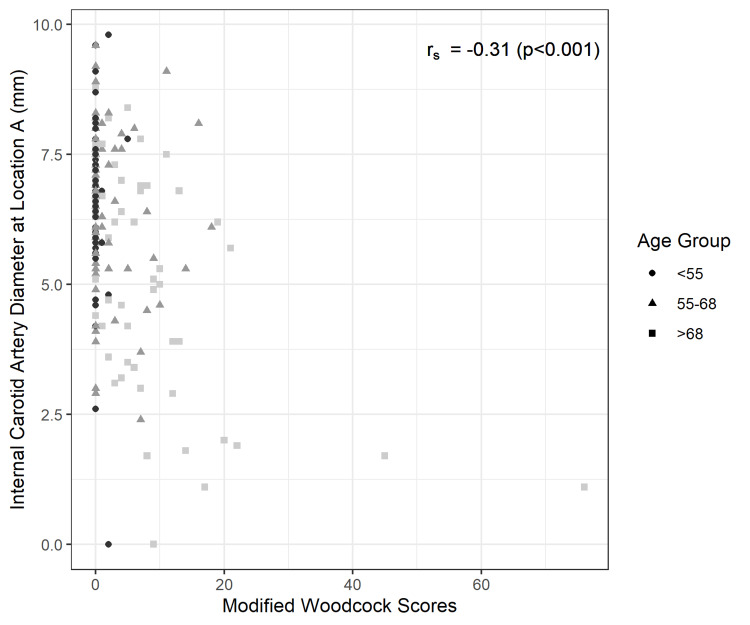
Scatter plot and Spearman’s rank correlation test: Modified Woodcock Scores and Internal Carotid Artery Diameters (mm)

**Figure 3:**
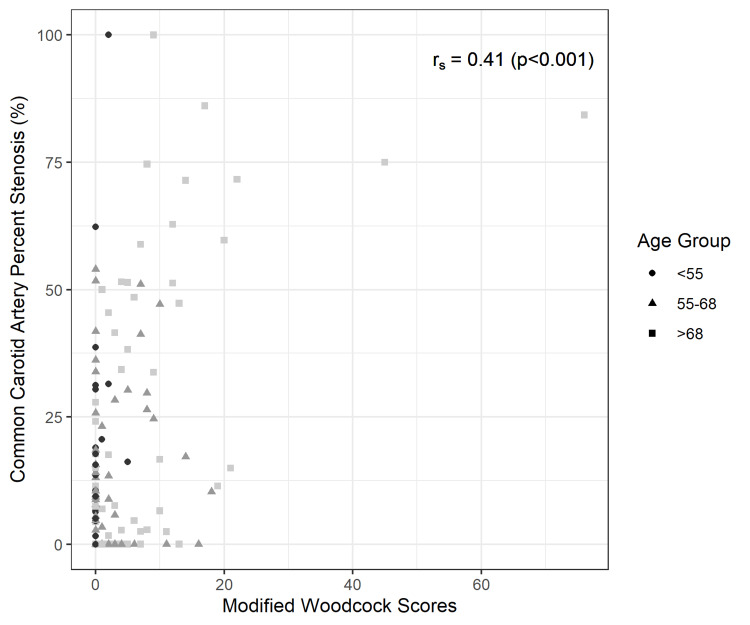
Scatter plot and Spearman’s rank correlation test: Modified Woodcock Scores and Common Carotid Artery Stenosis Ratios (%)

**Figure 4:**
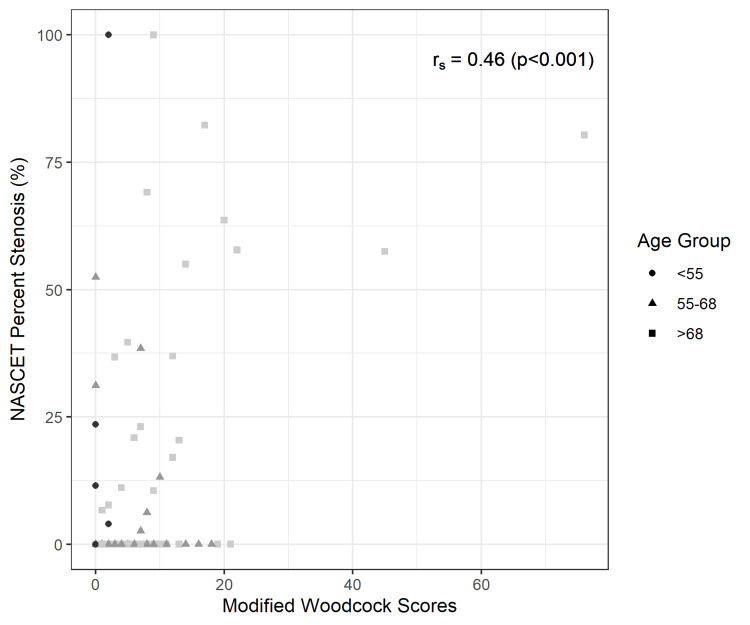
Scatter plot and Spearman’s rank correlation test: Modified Woodcock Scores and NASCET Stenosis Ratios (%) NASCET: North American Symptomatic Carotid Endarterectomy Trial

A Spearman’s rank correlation test demonstrated a significantly positive correlation between ICA stenosis and presence of calcification (p-values <0.001 in each of the three analyses). Figure 2 shows a negative monotonic correlation between modified Woodcock score and ICA absolute diameter. Figure 3 and Figure 4 show a positive monotonic correlation between modified Woodcock score and both CCA ratio and NASCET ratio percentages, respectively.

## Discussion

Extracranial ICA stenosis and intracranial ICA calcification have independently been identified as evidence of carotid disease [18]. The relationship between ICA stenosis and calcification is not well understood. Establishing a link between these two disease processes could help better understand cerebral artery disease.

In this pilot study, we measured extracranial ICA stenosis using absolute diameter, NASCET, and CCA methods and compared those results with discrete levels of intracranial ICA calcification quantified using the modified Woodcock visual scoring method. All three methods of measuring carotid stenosis supported the hypothesis that individuals with higher degrees of intracranial ICA calcification tended to have higher levels of extracranial ICA stenosis. These results help establish a link between extracranial stenosis and intracranial calcification in the ICA. As Doppler ultrasound is a more accessible imaging modality than a CT scan, this link can have clinical implications. The incorporation of routine monitoring of extracranial ICA stenosis can provide insight into the progression of intracranial calcification. Conversely, in individuals who undergo a CT scan, the incidental identification of intracranial calcification can raise suspicion for the presence of asymptomatic extracranial ICA stenosis. Incorporation of medical therapy and lifestyle modifications in patients with asymptomatic carotid artery disease has been previously shown to prevent stroke or cardiovascular disease [21]. Early recognition of vessel stenosis and subsequent intervention is crucial to improving patient outcomes.

Our results are consistent with the findings by Fisher et al. [18]. They showed that the degree of calcification increased with age and there is a positive correlation between carotid siphon calcification and atherosclerosis of the aorta, carotid, and cerebral arteries. The most significant correlation was made between severe calcification of the carotid siphon and advanced atherosclerosis of the carotid sinus as seen in our results.

Study limitations

One possible limitation of this study was the exclusion of CT images that were missing a bone scan. Due to the limited size of the database from which the CT scans were derived, a large prevalence of carotid stenosis was not guaranteed. Thus, the exclusion of brain images where carotid stenosis was identified, but no bone window was available, resulted in the exclusion of data points that could have influenced the results of this study. In addition, the impact of epidemiological factors, such as ethnicity, was not evaluated in this study. Future studies examining the potential risk factors could provide additional insight into epidemiological factors that may be associated with the development of atherosclerosis and ischemic stroke.

## Conclusions

The purpose of this study was to determine if there was a correlation between extracranial stenosis of the ICA and intracranial calcification of the ICA. It was concluded that individuals with intracranial ICA calcification were more likely to be older, have a smaller ICA diameter, and have a greater percent stenosis at the ICA than those without intracranial artery calcification (p<0.001 for all). This association can help prompt providers to order imaging studies to identify ICA stenosis or calcification that, otherwise, may have been missed. Identification of vessel narrowing prior to symptomology allows for early interventions and prevention of cerebrovascular disease progression. These results may help refocus interest in calcification in studies of cerebral vasculature and its correlation with extracranial carotid stenosis.
